# A Zic2/Runx2/NOLC1 signaling axis mediates tumor growth and metastasis in clear cell renal cell carcinoma

**DOI:** 10.1038/s41419-021-03617-8

**Published:** 2021-03-25

**Authors:** Chen-Yan Wu, Lei Li, Shi-Lu Chen, Xia Yang, Chris Zhiyi Zhang, Yun Cao

**Affiliations:** 1 Department of Pathology, State Key Laboratory of Oncology in South China and Collaborative Innovation Center for Cancer Medicine, Sun Yat-sen University Cancer Center, 510060 Guangzhou, China; 2 Department of Clinical Oncology, The University of Hong Kong, Hong Kong, China; 3 Key Laboratory of Functional Protein Research of Guangdong Higher Education Institutes and MOE Key Laboratory of Tumor Molecular Biology, Institute of Life and Health Engineering, College of Life Science and Technology, Jinan University, 510632 Guangzhou, China

**Keywords:** Tumour biomarkers, Renal cell carcinoma

## Abstract

Clear cell renal cell carcinoma (ccRCC) is one of the most common malignancies with rapid growth and high metastasis, but lacks effective therapeutic targets. Here, using public sequencing data analyses, quantitative real-time PCR assay, western blotting, and IHC staining, we characterized that runt-related transcription factor 2 (*Runx2*) was significantly upregulated in ccRCC tissues than that in normal renal tissues, which was associated with the worse survival of ccRCC patients. Overexpression of *Runx2* promoted malignant proliferation and migration of ccRCC cells, and inversely, interfering *Runx2* with siRNA attenuates its oncogenic ability. RNA sequencing and functional studies revealed that Runx2 enhanced ccRCC cell growth and metastasis via downregulation of tumor suppressor nucleolar and coiled-body phosphoprotein 1 (*NOLC1*). Moreover, increased Zic family member 2 (*Zic2*) was responsible for the upregulation of Runx2 and its oncogenic functions in ccRCC. Kaplan–Meier survival analyses indicated that ccRCC patients with high Zic2/Runx2 and low NOLC1 had the worst outcome. Therefore, our study demonstrates that Zic2/Runx2/NOLC1 signaling axis promotes ccRCC progression, providing a set of potential targets and prognostic indicators for patients with ccRCC.

## Introduction

Renal cell carcinoma (RCC), also known as renal adenocarcinoma, is a common malignant tumor arising from the renal tubular epithelial system. In 2019, the cancer statistics in United States showed an estimated 73,820 new diagnoses and 14,770 deaths for RCC. The incidence and mortality of RCC are increasing every year^1^. There are main causes of RCC, such as smoking, obesity, cadmium exposure, the use of chronic analgesics, cystic kidney disease, and certain genetic syndromes^2^. According to molecular characteristics and histological classification, clear cell renal cell carcinoma (ccRCC) is the most common histological subtype in RCC accounting for nearly 80% of RCC patients^3^. Although patients with early-stage ccRCC can obtain a well therapeutic effect through nephrectomy or nephrectomy-preserving tumor resection, there are still many cases with local recurrence or distant metastasis. Especially metastatic ccRCC, the efficacy of traditional chemical drugs is insignificant because of the high drug resistance of ccRCC cells^4,5^. Therefore, investigating the key oncogenes involved in the aberrant proliferation and metastasis of ccRCC is a valuable goal toward revealing the mechanisms of cancer progression and identifying new therapeutic targets for ccRCC treatment.

Runt-related transcription factor (Runx) proteins, including Runx1, Runx2, and Runx3, are known as transcription factors in the physiological and pathological processes of the body. Runx2 as a member of the Runx family is widely involved in bone formation and osteoblast differentiation^6^. Furthermore, Runx2 effectively prevented apoptosis of intestinal epithelial cells during the development of Crohn’s disease^7^. In the last decade, Runx2 has been shown to be involved in the regulation of cancer progression with dual functions of oncogenic potential and tumor suppressor abilities^8,9^. Transcriptional inhibition of Runx2 was negatively correlated with aggressive clinicopathological outcomes, whereas nuclear location of Runx2 promoted metastasis in prostate cancer^10^. Overexpression of Runx2 suppressed osteosarcoma cell growth in vitro^11^. Runx2 could inhibit p53-dependent apoptosis through the functional collaboration with HDAC6 in response to DNA damage^12^. In addition, increasing evidences suggested an aggressive role of Runx2 in various cancers. For instance, high expression of Runx2 was significantly correlated with larger tumor size, lymph node metastasis, and shorter postoperative survival time of patients with non-small cell lung cancer^13^. Repression of Runx2 transcriptional activity blocked the proliferation, migration, and invasion of epithelial ovarian carcinoma cells^14^. In-depth studies revealed that Runx2 promoted cancer progression by inducing the epithelial–mesenchymal transition (EMT) progress in breast cancer^15^, thyroid carcinoma^16^, and hepatocellular carcinoma (HCC)^17^. Therefore, dysregulation of Runx2 modulates tumor cell proliferation and metastasis, and revealing the underlying mechanism is urgent in the future development of individual treatments against different types of cancer. However, the expression and role of Runx2 in ccRCC are, as yet, unclear.

In the present study, we clearly characterized the expression and prognosis prediction of Runx2 in ccRCC, and found that aberrant overexpression of Runx2 was significantly associated with poor survival of ccRCC patients. Functional and mechanism studies revealed that Runx2 was upregulated by Zic family member 2 (Zic2) and enhanced ccRCC cell proliferation and migration via transcriptional inhibition of tumor suppressor nucleolar and coiled-body phosphoprotein 1 (NOLC1). Therefore, our findings clearly demonstrate that dysregulation of Zic2/Runx2/NOLC1 signaling promotes ccRCC progression.

## Materials and methods

### Clinical samples and cell lines

Human ccRCC and normal renal tissues were obtained from ccRCC patients without preoperative chemoradiotherapy treatment at Sun Yat-sen University Cancer Center (Guangzhou, China). Tissue microarray containing 301 paired ccRCC and normal renal tissues was constructed in our laboratory, and all cases were collected between 2004 and 2012 with integrated clinicopathologic and follow-up data. This study was approved by the institutional research ethics committee of Sun Yat-sen University Cancer Center (Guangzhou, China). Human immortalized renal epithelial cell 293T and ccRCC cell lines ACHN, 786-O, SKRC39, and CAKI-1 were preserved in our laboratory. All cells were cultured with DMEM (Gibco) supplemented with 10% fetal bovine serum (FBS, Gibco) at 37 °C with 5% CO_2_. When the cells reached 80–90% confluence, digestion and passage were performed with trypsinization.

### Plasmid construction, RNA interference, and cell transfection

The coding sequence of Runx2 was obtained by reverse transcription PCR (RT-PCR) and cloned into pZM02 vector. The promoter of NOLC1 was also amplified by RT-PCR and cloned into pGL4.10 luciferase reporter vector. The expressions of Zic2, Runx2, and NOLC1 were silenced with siRNA oligonucleotides (Table S1). Scramble RNA was used as a negative control. The culture medium was changed to fresh DMEM with 10% FBS 24 h before transfection. The plasmids and siRNA oligonucleotides were transfected into cancer cells with Lipofectamine 2000 (50 nM, Invitrogen). Transfected cells were cultured at 37 °C with 5% CO_2_ for 6 h, and then the culture medium was replaced by fresh normal medium. The expression levels of target genes were confirmed with quantitative real-time PCR (qRT-PCR) and western blot at 24 or 36 h after transfection, respectively.

### RNA extraction and qRT-PCR

Total RNA from fresh tissues or cell lines was extracted with TRIzol Reagent (Invitrogen). Note that fresh ccRCC and normal renal tissues were frozen with liquid nitrogen and grinded until powdered before lysis with TRIzol. Complementary DNA was synthesized with reverse transcriptase (Takara) according to the manufacturer’s instruction. The mRNA expression levels of genes were analyzed by qRT-PCR using FastStart Universal SYBR Green Master (Roche) and a Real-Time PCR Detection System (Roche); 18S rRNA was tested as an internal control. The relative expression level (defined as fold change) of the target gene (2^−ΔΔCt^) was normalized to the endogenous 18S reference (ΔCt). The primers used were listed in Table S2.

### Western blot

Western blot analysis was performed according to the standard protocol^18^. Human fresh ccRCC/normal renal tissues and cells were lysed with RIPA lysis buffer (Cell Signaling Technology) supplemented with cocktail protease inhibitor (Roche) in ice for 30 min. After centrifugation with 12,000 r.p.m. for 30 min at 4 °C, the supernatant was collected for protein concentration quantification. Protein lysates were separated by 5–10% SDS-PAGE and then transferred to PVDF membrane (Millipore). To prevent non-specific binding, the membrane was blocked with 5% bovine serum albumin in TBST buffer for 1 h at room temperature and incubated with primary antibodies against Zic2 (Abcam, #ab150404), Runx2 (Cell Signaling Technology, #12556), and NOLC1 (Abcam, #ab184550) overnight at 4 °C. β-Actin (Cell Signaling Technology, #4970) was used as a loading control. After rinsing three times with TBST buffer for 5 min each time, the membrane was incubated with HRP-conjugated secondary antibodies for 2 h at room temperature. The target proteins were visualized with Super ECL Detection Reagent (Thermo Fisher Scientific).

### Immunohistochemical staining

Immunohistochemical (IHC) staining was performed as described previously^19,20^. In brief, paraffin tissue sections were deparaffinized by xylene, rehydrated using graded ethanol, and blocked with 3% hydrogen peroxide for eliminating endogenous peroxidase activity. After antigen retrieval and non-specific binding blockade, tissue sections were incubated with primary antibodies against Zic2 (Abcam, #ab150404), Runx2 (Cell Signaling Technology, #12556), and NOLC1 (Abcam, #ab184550). HRP-conjugated secondary antibody and DAB substrates were used for staining. The representative images were captured with light microscope (Olympus). IHC staining score system and ROC curves were applied to distinguish the cases with high or low expression of target protein. The percentage of positive cells was scored as 1, <25%; 2, 25–50%; 3, 50–75%; 4, >75%. The intensity of IHC staining was scored as 0, negative; 1, weak; 2, moderate; 3, strong. Total score = positive percentage × intensity.

### MTT cell growth assay

Transfected and control ccRCC cells (3000 cells/well) were seeded into 96-well culture plate, and cultured at 37 °C with 5% CO_2_. After 24 h, the culture medium was replaced by new medium supplemented with MTT (100 μg/ml). Four hours after culture, the culture supernatant was removed gently, and changed into DMSO (150 μl/well). Culture plate was put on a shaker at a low speed for 10 min to fully dissolve the crystals. The absorbance values of each well were measured by Automatic Microplate Reader at 570 nm.

### Cell migration assay

After 24 h from transfection, cancer cells were treated with 0.25% trypsin, and resuspended with serum-free medium. Cell number was measured with cell counting chamber. Cancer cells (2 × 10^4^/well) were planted into Transwell chamber (Corning) in a 24-well plate. Culture medium containing 20% FBS was added to the lower chamber of the Transwell. The plate was cultured at 37 °C with 5% CO_2_ for 24 h, and cells were fixed with methyl alcohol for 15 min, and then stained with 0.1% crystal violet solution for 20 min at room temperature. The Transwell was washed three times with PBS. Cancer cells in the upper part of the Transwell were removed by cotton swabs. Five to eight fields of cells were randomly selected on an inverted microscope (Olympus) and then statistically analyzed.

### Foci formation assay

Cancer cells (1000/well) were seeded into 6-well culture plates, and cultured at 37 °C with 5% CO_2_. The culture medium was changed every 3 days for 2 weeks. Next, the culture medium was removed, and the cells were washed three times with PBS, fixed with methyl alcohol for 15 min, and stained with 0.1% crystal violet solution for 20 min at room temperature. After washing with PBS, the images of foci were captured with a scanner, and then the number of foci containing more than ten cells was counted.

### In vivo xenograft assay

All animal procedures were approved by Animal Ethics Committee at Sun Yat-sen University Cancer Center (Guangzhou, China). Five-week-old male BALB/c nude mice were purchased from the Guangdong Medical Laboratory Animal Center (Guangzhou China). Mice of the same age and sex were randomly assigned to experimental groups. For tumor formation in vivo, 1 × 10^6^ ACHN and 786-O cells after knockdown of Runx2 were resuspended with 100 μl of PBS and subcutaneously injected into the back of nude mice, respectively (4 mice/group). The length (*L*) and width (*W*) of tumor were measured every 3 days with calipers, and the tumor volumes were calculated as volume (mm^3^) = *L* × *W*^2^ × 0.5. The mice were sacrificed 35 days after inoculation, and the tumor weights were measured with electronic scales. For the lung metastasis in nude mice, 5 × 10^5^ 786-O cells with or without Runx2 knockdown were suspended in 100 μl of PBS and injected into the tail vein of nude mice (six mice for each group). The mice were sacrificed 6 weeks after cell injection, and lung tissues were harvested and fixed with formaldehyde solution for hematoxylin–eosin staining. The lung metastatic nodules were counted under light microscope (Olympus).

### Statistics

SPSS 19.0 software (Chicago, IL) was used for all data statistics. All the key experiments were repeated at least three times. The comparison between the two groups of continuous data is conducted by independent Student’s *t-*test. Log-rank test was used for Kaplan–Meier survival analysis. Survival curves based on The Cancer Genome Atlas (TCGA) database were obtained from GEPIA (http://gepia2.cancer-pku.cn/). *P* value <0.05 was considered statistically significant.

## Results

### High expression of Runx2 is related to the worse outcome of ccRCC patients

**Fig. 1:**
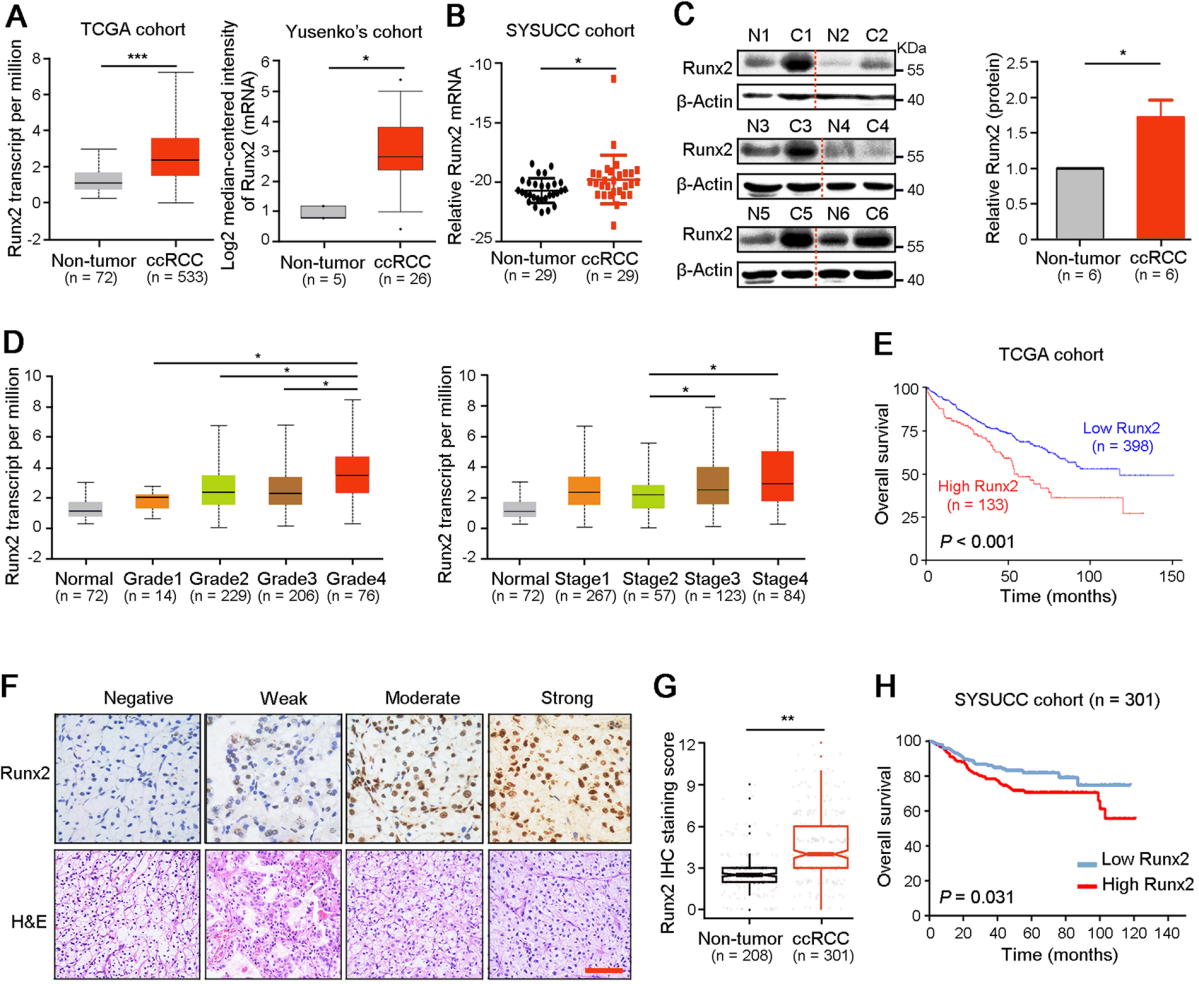
High expression of Runx2 is related to the worse outcome of ccRCC patients. **A** TCGA and Oncomine (Yusenko’s cohort) database analyses showed the high level of Runx2 in ccRCC tissues than that in non-tumor renal tissues. **B** qRT-PCR was used to test the mRNA level of Runx2 in ccRCC and paired non-tumor renal tissues (*n* = 29). **C** Western blot analyses of the expression of Runx2 in ccRCC and corresponding non-tumor renal tissues at protein level (*n* = 6). β-Actin was also tested as a loading control. **D** High expression of Runx2 was associated with the poorer tumor grade and patient stage in ccRCC based on the TCGA dataset analysis. **E** Survival cures from TCGA cohort showed that ccRCC patients with high Runx2 had a short overall survival (*P* < 0.001). **F** Representative images of IHC staining demonstrated the different expression level of Runx2 in ccRCC tissues. Scale bar, 40 μm. **G** Score analysis of IHC staining showed the high expression of Runx2 in ccRCC tissues (*n* = 208) than that in non-tumor renal tissues (*n* = 301). **H** Kaplan–Meier survival analysis indicated that high expression of Runx2 was associated with poorer survival of patients with ccRCC (*P* = 0.031). In all panels, *, *P* < 0.05; **, *P* < 0.01; ***, *P* < 0.001.

To investigate the expression and role of Runx2 in ccRCC progression, we analyzed the expression of Runx2 in ccRCC and normal renal tissues in TCGA and Oncomine (Yusenko’s cohort) databases. Results showed the upregulation of Runx2 in ccRCC tissues at mRNA level compared to normal renal tissues (Fig. 1A). qRT-PCR and western blot confirmed the high expression of Runx2 in ccRCC tissues than that in paired normal renal tissues (Fig. 1B, C). Next, correlations analyses of Runx2 level and clinicopathological characteristics based on TCGA dataset suggested that high expression of Runx2 was associated with poorly differentiated grade and advanced stage of ccRCC (Fig. 1D). Survival analysis using TCGA clinical data showed that ccRCC patients with high expression of Runx2 had shorter overall survival and disease-free survival than patients with low level of Runx2 (*P* < 0.001, Figs. 1E and S1). Furthermore, IHC staining in ccRCC tissue array was used to confirm the expression level and prognostic significance of Runx2 (Fig. 1F). Results found that protein Runx2 was significantly upregulated in ccRCC compared to normal renal tissues (*P* < 0.01, Fig. 1G). In addition, ccRCC patients with high or low expression of Runx2 were distinguished with ROC curve analysis based on IHC staining scores. Kaplan–Meier survival analysis showed that high expression of Runx2 was correlated with adverse overall survival for ccRCC patients (*P* = 0.031, Fig. 1H). These findings suggested that Runx2 was a hazardous factor of ccRCC progression.

### Overexpression of Runx2 promotes ccRCC cell proliferation and migration

**Fig. 2:**
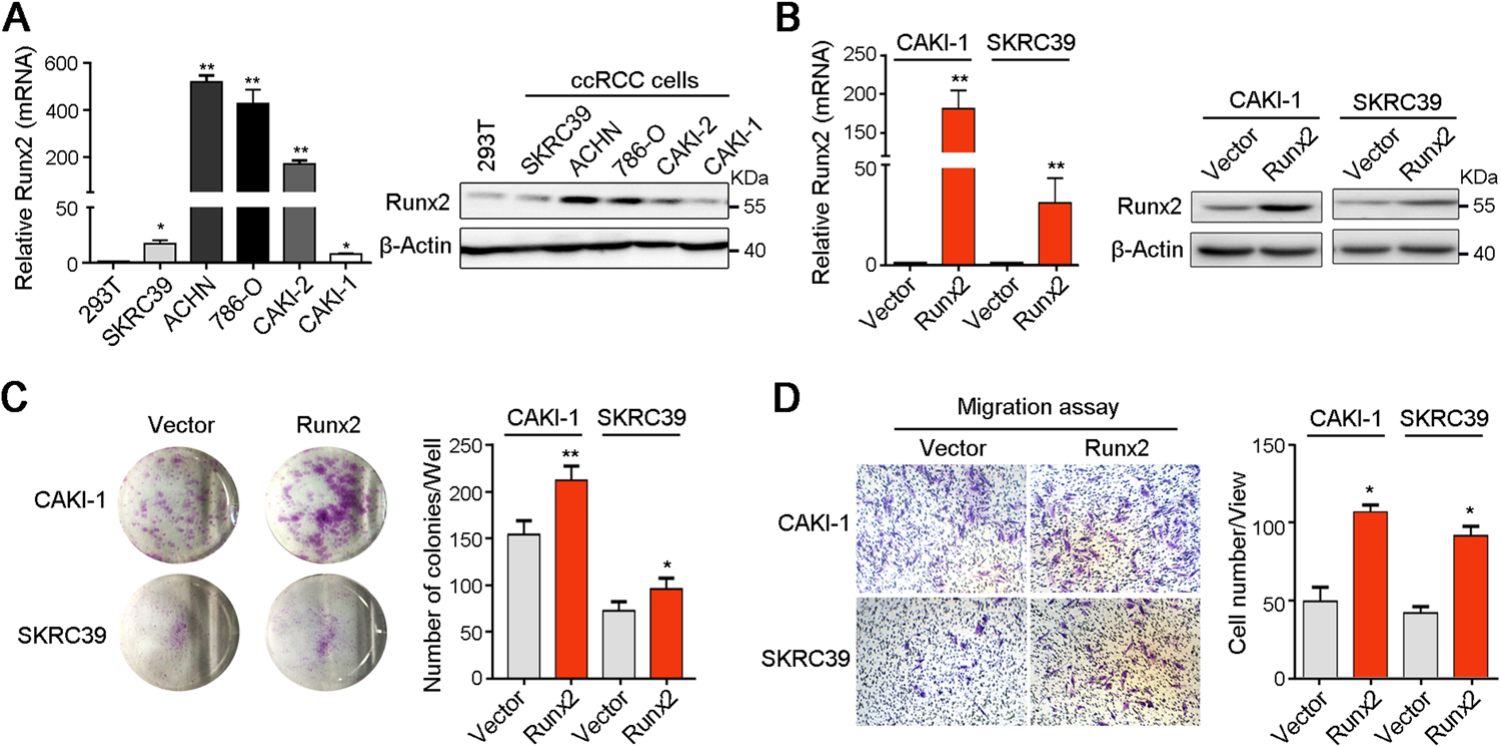
Exogenous overexpression of Runx2 enhances the proliferation and migration of ccRCC cells. **A** The expression level of Runx2 in one human immortalized renal epithelial cell and five ccRCC cells was analyzed by qRT-PCR and western blot analyses, respectively. β-Actin was also tested as a loading control. **B** Plasmid-mediated transfection was used to overexpress the Runx2 in CAKI-1 and SKRC39 cells, and the expression level of Runx2 was confirmed by qRT-PCR and western blot analyses. **C** Foci formation assay showed that overexpression of Runx2 promoted ccRCC cells growth. **D** Migration assay suggested that the migration ability of ccRCC cells was increased after overexpression of Runx2, compared to control cells. In all panels, *, *P* < 0.05; **, *P* < 0.01.

Next, qRT-PCR and western blot analyses were used to test the expression of Runx2 in one human immortalized renal cell 293T and five ccRCC cell lines at mRNA and protein levels, respectively. Results showed the high expression of Runx2 in ccRCC cells than that in 293T cell (Fig. 2A). To explore the function of Runx2 in ccRCC, two ccRCC cell lines CAKI-1 and SKRC39 with relative low expression of Runx2 were transfected with plasmid containing the coding sequence of Runx2, and overexpression of Runx2 was confirmed by qRT-PCR and western blot analyses (Fig. 2B). The role of Runx2 in cell growth regulation was evaluated by foci formation assay in vitro. Compared to vector-transfected cells, enhanced Runx2 significantly increased the frequency of foci formation in CAKI-1 and SKRC39 cells (Fig. 2C). In addition, Transwell migration assay showed that increased Runx2 could also enhance the migration ability of ccRCC cells (Fig. 2D). These evidences indicated the aggressive role of Runx2 in ccRCC.

### Knockdown of Runx2 suppresses the growth and metastasis of ccRCC cells

**Fig. 3:**
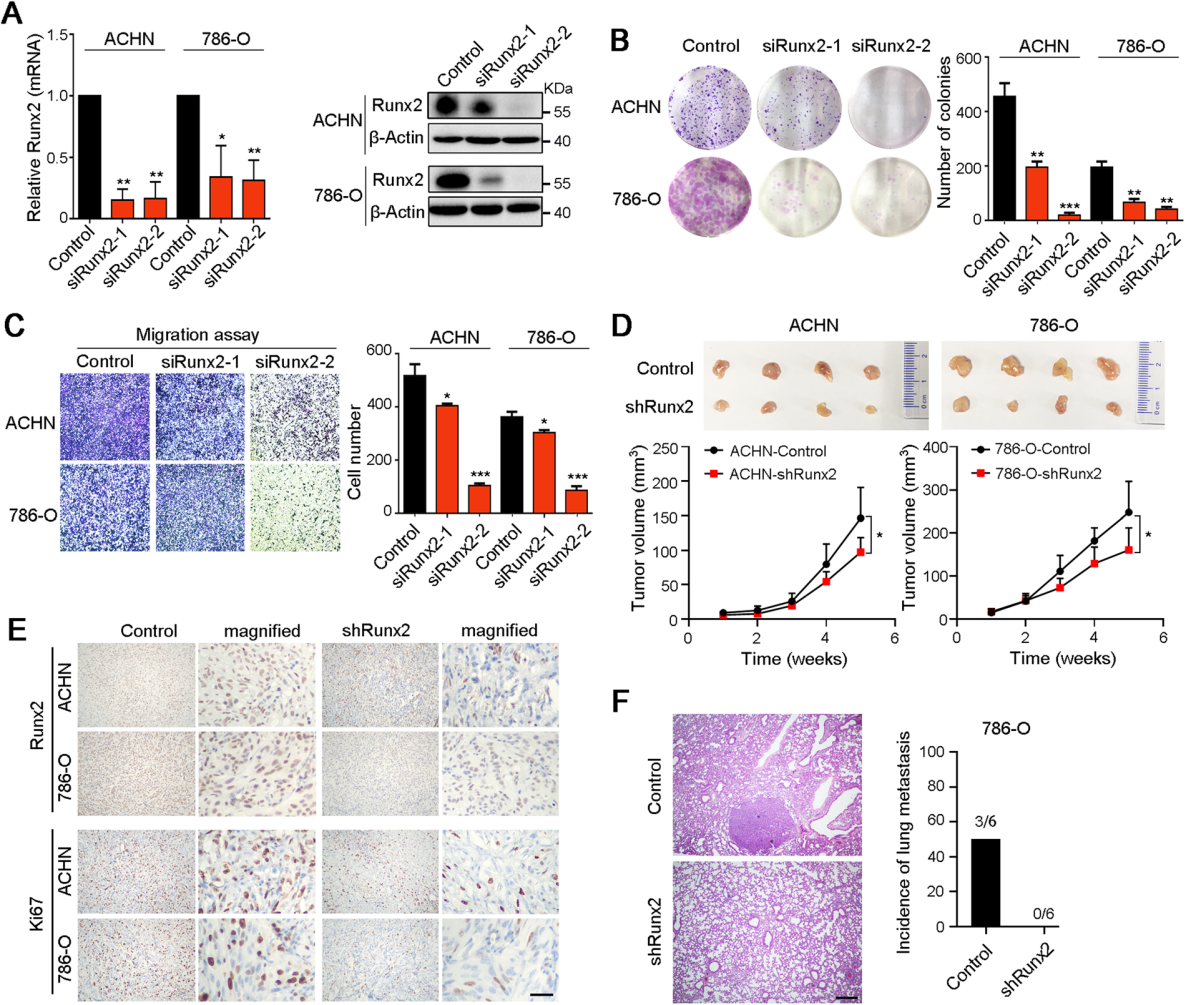
Knockdown of Runx2 attenuates the growth and metastasis of ccRCC cells. **A** Two siRNAs were used to downregulate the expression of Runx2 in ACHN and 786-O cells, and the knockdown of Runx2 was confirmed by qRT-PCR and western blot analyses, respectively. **B** Interference of Runx2 decreased the foci formation frequencies of ccRCC cells. **C** Transwell migration assay was performed to explore the effect of Runx2 on the migration ability of ccRCC cells. **D** ACHN and 786-O cells after knockdown of Runx2 were subcutaneously injected into nude mice, and the tumor volumes were measured every week (*n* = 4 mice per group). **E** IHC staining with antibodies against Runx2 and Ki67 in xenograft tumors derived from ACHN and 786-O cells with or without Runx2 silencing. Scale bar, 25 μm. **F** Nude mice were treated by tail intravenous injection of 786-O cells with or without Runx2 knockdown. Hematoxylin–eosin staining confirmed the lung metastasis, and the incidence of metastasis was indicated in right panel (*n* = 6 mice per group). Scale bar, 50 μm. In all panels, *, *P* < 0.05; **, *P* < 0.01; ***, *P* < 0.001.

Inversely, the upregulation of Runx2 in two ccRCC cell lines ACHN and 786-O were interfered with two siRNAs targeting Runx2, which was evaluated by qRT-PCR and western blot analyses (Fig. 3A). Next, the in vitro and in vivo functional studies were performed to analyze the effect of Runx2 knockdown on ccRCC cell growth and metastasis. Firstly, downregulation of Runx2 in ACHN and 786-O cells could inhibit foci formation (Fig. 3B) and cell migration (Fig. 3C). ACHN and 786-O cells with knockdown of Runx2 were subcutaneously injected into nude mice, and the tumor volume was measured every week. Results showed that tumors derived from ACHN and 786-O cells with knockdown of Runx2 were growing slowly, compared to tumors originated from the control cells (Fig. 3D). IHC staining also confirmed the decreased expression of Runx2 and Ki67 in xenograft tumors with Runx2 silencing (Fig. 3E). Moreover, lung metastasis assay in nude mice was performed by tail intravenous injection of 786-O cells with or without Runx2 knockdown. Results indicated that knockdown of Runx2 inhibited ccRCC cell metastasis (Fig. 3F). Therefore, Runx2 plays a vital role in ccRCC growth and metastasis.

### Runx2 accelerates ccRCC progression by inhibiting the expression of NOLC1

**Fig. 4:**
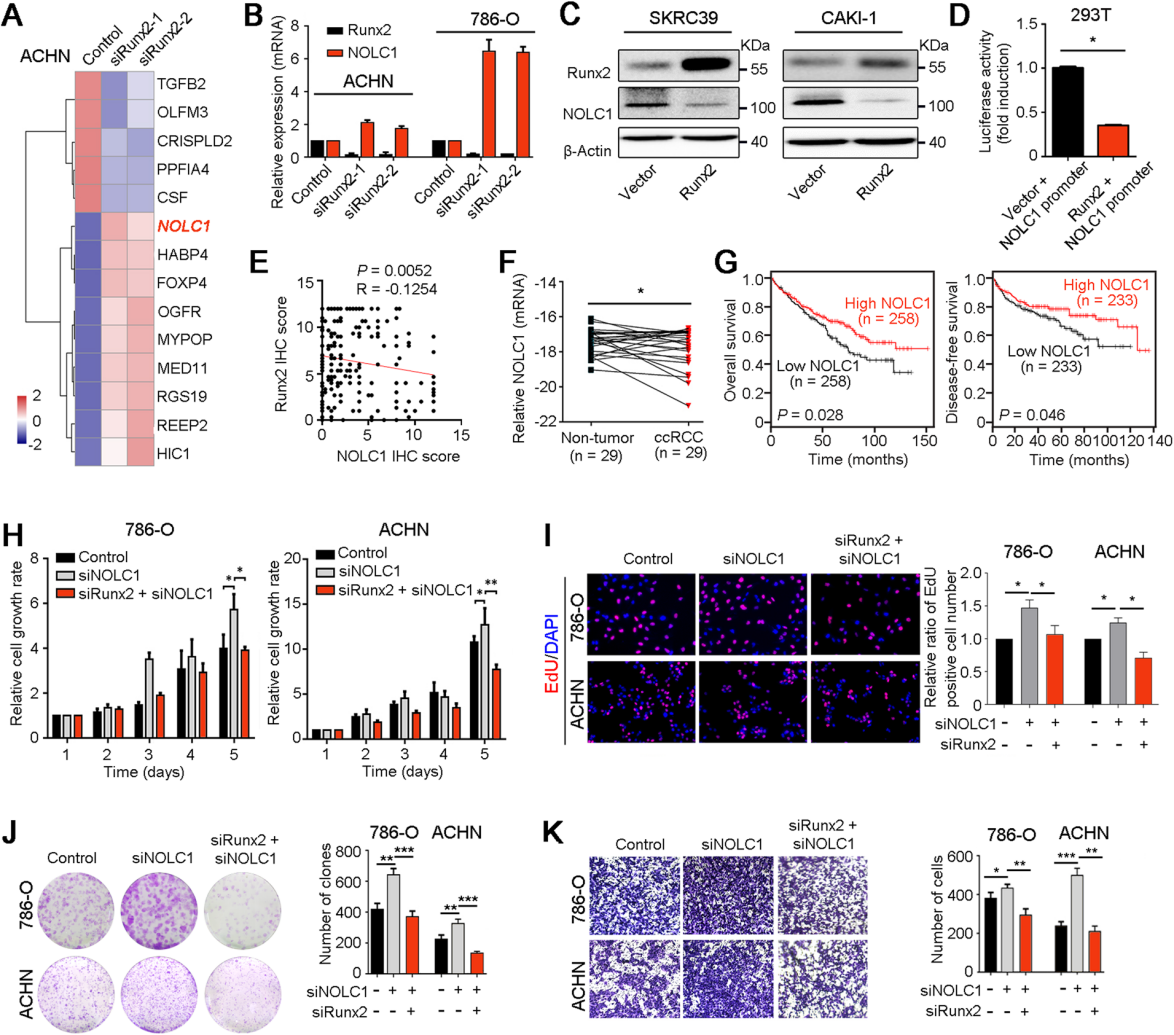
NOLC1 mediates the regulation of Runx2 in ccRCC cell growth and metastasis. **A** RNA-sequencing was performed on ACHN cells transfected with two siRNA targeting Runx2, and the genes with significant changes were indicated by heat map. **B** The mRNA expressions of Runx2 and NOLC1 in ACHN and 786-O cells after knockdown of Runx2 were analyzed by qRT-PCR. **C** Western blot analysis showed the downregulation of NOLC1 in SKRC39 and CAKI-1 cells after overexpression of Runx2. β-Actin was also tested as a loading control. **D** Luciferase report assay showed that high expression of Runx2 inhibited the transcription of NOLC1. **E** IHC staining analysis in ccRCC tissue array showed the negative correlation between the expressions of Runx2 and NOLC1. **F** qRT-PCR was used to analyze the expression level of NOLC1 in ccRCC and corresponding normal renal tissues at mRNA level. **G** Survival analyses of ccRCC patients with different expression level of NOLC1 based on TCGA datasheet. **H**–**J** MTT test (**H**), EdU incorporation (**I**), and foci formation (**J**) assays were performed to analyze the proliferation of 786-O and ACHN cells after knockdown of NOLC1 or/and Runx2, respectively. **K** The migration ability of ccRCC cells after interference of NOLC1 or/and Runx2 was analyzed by Transwell migration assay. In all panels, *, *P* < 0.05; **, *P* < 0.01; ***, *P* < 0.001.

To explore the mechanism of Runx2 promoting ccRCC growth and metastasis, we performed RNA sequencing of ACHN cell with or without Runx2 silencing (Fig. 4A). qRT-PCR and western blot analyses confirmed that NOLC1 was significantly upregulated after knockdown of Runx2 in ACHN and 786-O cells (Fig. 4B) and inversely downregulated when Runx2 was overexpressed in SKRC39 and CAKI-1 cells (Fig. 4C). The promoter of NOLC1 was cloned into luciferase report vector and then was co-transfected with Runx2-expressed plasmid into 293T cell. Luciferase activity assay showed that overexpression of Runx2 attenuated the transcription of NOLC1 (*P* < 0.05, Fig. 4D). Moreover, IHC staining analysis showed the negative correlation between the expressions of Runx2 and NOLC1 in ccRCC tissues (Fig. 4E). There is a binding site of Runx2 in the upstream region (−107 – −102 nt) of the transcription start site of NOLC1 (Fig. S2A, B). ChIP-qPCR assay confirmed that Runx2 bound to the promoter of NOLC1 in ACHN and 786-O cells (Fig. S2C). These data suggested that NOLC1 expression was negatively regulated by Runx2 in ccRCC.

NOLC1 as a protein chaperone for shuttling between the nucleolus and cytoplasm participates in the regulation of rRNA transcription^21^. Recent study reported that NOLC1 expression was decreased in HCC tissue, and ectopic expression of NOLC1 inhibited tumor growth in mouse by repressing the proliferation of HCC cells^22^, which suggests the tumor suppressive role of NOLC1. TCGA database analysis showed the high expression of NOLC1 in normal renal tissues, compared to ccRCC tissues (*P* < 0.01, Fig. S2D). We also confirmed that NOLC1 was lowly expressed in ccRCC tissues than that in normal renal tissues at mRNA and protein levels analyzed by qRT-PCR (Fig. 4F) and IHC staining (Fig. S2E). Moreover, survival analyses using TCGA data indicated that ccRCC patients with low level of NOLC1 had short overall and disease-free survival than whose with high expression of NOLC1 (Fig. 4G). Importantly, in vitro functional analyses showed that silencing of NOLC1 could rescue the inhibition of cell growth and migration induced by Runx2 knockdown in 786-O and ACHN cells (Fig. 4H–K). Therefore, Runx2 promoted ccRCC progression via downregulation of tumor suppressor NOLC1.

### Zic2 upregulates Runx2 to promote ccRCC cell growth and metastasis

**Fig. 5:**
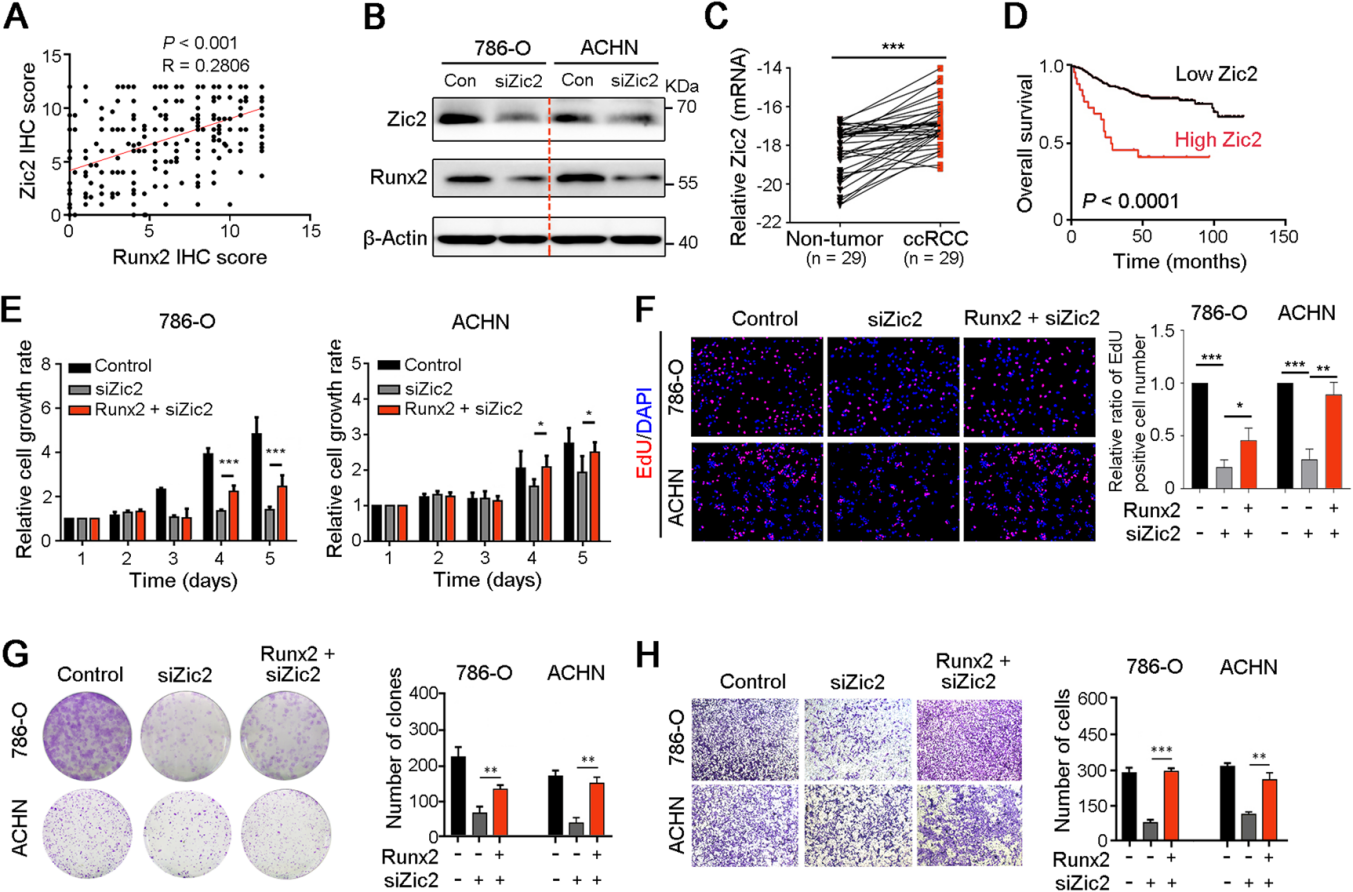
Zic2 upregulates the expression of Runx2 promoting ccRCC cell growth and metastasis. **A** IHC staining analysis in ccRCC tissue array showed the positive correlation of Zic2 and Runx2 expressions. **B** Western blot analysis showed the downregulation of Runx2 in 786-O and ACHN cells after knockdown of Zic2. β-Actin was also tested as a loading control. **C** qRT-PCR was used to analyze the expression level of Zic2 in ccRCC and paired normal renal tissues (*n* = 29). **D** Survival curves based on IHC staining indicated the poorer outcome of ccRCC patients with high expression of Zic2 than those with low level of Zic2 (*P* < 0.0001). **E**–**G** MTT test (**E**), EdU incorporation (**F**), and foci formation (**G**) assays were performed to analyze the proliferation of 786-O and ACHN cells after knockdown of Zic2 or/and Runx2 overexpression, respectively. **H** The migration ability of ccRCC cells after interference of Zic2 or/and overexpression of Runx2 was tested by Transwell migration assay. In all panels, *, *P* < 0.05; **, *P* < 0.01; ***, *P* < 0.001.

The above data have demonstrated that high expression of Runx2 aggravated ccRCC progression, but the regulation of Runx2 expression in ccRCC was unclear. Promoter methylation analysis with TCGA data showed that the methylation level of Runx2 in ccRCC and normal renal tissues has no significant difference (Fig. S3A). In addition, copy number alterations were not responsible for the upregulation of Runx2 in ccRCC tissues based on TCGA database analysis (Fig. S3B). TCGA data analysis and IHC staining showed the positive correlation between Zic2 and Runx2 levels in ccRCC (Figs. 5A and S4A, B). Silencing Zic2 with siRNA in 786-O and ACHN cells reduced the expression of Runx2 at mRNA and protein levels (Figs. 5B and S4C), which indicated that Runx2 expression in ccRCC was regulated by Zic2.

Zic2 has been shown to play important roles in carcinogenesis. Knockdown of Zic2 inhibited the proliferation, migration, and invasion of breast cancer cells^23^. Restoration of Zic2 expression promoted the proliferation and reduced the apoptosis of AML cells^24^. High expression of Zic2 was related to the worse overall survival of patients with ccRCC^25^. Here, qRT-PCR (Fig. 5C), TCGA analysis (Fig. S4D), and IHC staining (Fig. S4E) showed the upregulation of Zic2 in ccRCC tissues. Survival analysis suggested that high expression of Zic2 was associated with the worse outcome of patients with ccRCC (*P* < 0.0001, Fig. 5D). In addition, western blotting showed the high expression of Zic2 and low expression of NOLC1 in ccRCC cells (Fig. S4F). Previous study has proved that enhanced NOLC1 inhibited cell growth by perturbing rRNA synthesis^22^. We confirmed the downregulation of 45S pre-rRNA in 786-O cells after knockdown of Zic2 and Runx2 by qRT-PCR (Fig. S5). Knockdown of Zic2 significantly inhibited ccRCC cell proliferation and migration and attenuated the oncogenic ability of Runx2 in 786-O and ACHN cells (Fig. 5E–H). In conclusion, dysregulation of Zic2/Runx2/NOLC1 signaling enhanced ccRCC progression by promoting cell growth and metastasis.

### Aberrant Zic2/Runx2/NOLC1 signaling predicted the survival of ccRCC patients

IHC staining analyses with antibodies against Zic2, Runx2, and NOLC1 in ccRCC tissue microarray were respectively performed to synthetically evaluate the survival of ccRCC patients based on the expression level of Zic2/Runx2/NOLC1 axis. ccRCC patients were distinguished into high or low expression groups based on the ROC curve analyses of IHC staining scores. Results of Kaplan–Meier survival analyses showed that co-overexpression of Zic2 and Runx2 in ccRCC tissues predicated the worse prognosis of ccRCC patients, compared to other groups (*P* < 0.001, Fig. S6A). Finally, multi-survival curves showed that ccRCC patients with high Zic2/Runx2 and low NOLC1 had the worst overall survival (*P* < 0.001, Fig. S6B). Therefore, Zic2/Runx2/NOLC1 signaling axis was a promising prognostic factor for patients with ccRCC.

## Discussion

Runx genes can behave as oncogenes or tumor suppressor genes exhibiting dual and contradictory functions in cancer progression. The inconsistent expression level and pro-/anti-tumorigenic roles of Runx2 in different cancers have been investigated by many research groups^26–29^. In this study, we proved that the expression of Runx2 was aberrantly increased in ccRCC, which promoted cell proliferation and metastasis and predicted the worse survival of patients with ccRCC. Therefore, our study confirms the aggressive role of Runx2 in ccRCC, and it may service as a promising therapeutic target for ccRCC treatment.

Recent studies have partially revealed the underlying secrets involved in the contradictory functions of Runx2 in ccRCC. Low expression of Runx2 was associated with aggressive clinicopathological characteristics for patients with prostate cancer, but most patients with metastatic disease were Runx2 nuclear staining positive, which suggested that the nuclear location of Runx2 was related with metastasis in prostate cancer^10^. In the present study, we also found the significant nuclear location of Runx2 in ccRCC cells by IHC staining. Therefore, distinct cellular localization of Runx2 may be responsible for the different functions of Runx2 during cancer progression. Otherwise, one study showed that the growth suppressive activity of Runx2 was normally inactivated in part by protein destabilization, which permited cell cycle progression beyond the G1/S phase transition, and Runx2 was upregulated again after mitosis in human osteosarcoma cells. Accumulation of Runx2 in excess of its pre-established level will inhibit cell proliferation, and balancing protein level of Runx2 exhibits its putative oncogenic functions^11^. Thus, the therapeutic control of Runx2 expression can change its oncogenic role into tumor suppressive function, providing a novel treatment strategy^30^. In addition, whether Runx2 is an activator or repressor in cancer progression may depend on the interacting coactivators or corepressors recruited by Runx2 at target promoters^31^. In this study, we proved that Runx2 as an oncogene enhanced proliferation and migration of ccRCC cells by inhibiting the transcription level of tumor suppressor NOLC1, revealing a novel oncogenic mechanism of Runx2 in cancer progression.

RNA sequencing was used to analyze the target genes of Runx2 in ccRCC cells, and we found that gene NOLC1 expression was significantly downregulated after silencing of Runx2. Previous reports have demonstrated that the transcription of NOLC1 in cancers was regulated by transcription factors YAP1 (ref. ^32^) and p53 (refs. ^33,34^). Our findings revealed that Runx2 functioned as a novel transcription repressor of NOLC1 in ccRCC. In addition, cell proliferation and migration assays also confirmed that regression of NOLC1 was responsible for the oncogenic function of Runx2 in ccRCC cells. NOLC1, also known as NOPP140, was a nucleolar chaperone protein participating in nucleolus construction and rRNA synthesis^35^. The expression and role of NOLC1 in cancer have not been clearly investigated. Increasing evidences suggested that NOLC1 played a tumor suppressive role by inducing cell-cycle arrest^36,37^. Otherwise, NOLC1 as a cell senescence-associated gene was usually downregulated in HCC cells^22^ and CD26-positive quiescent leukemia stem cells^38^. In addition, NOLC1 was also confirmed to be overexpressed in multidrug-resistant lung cells^39^. Here, we showed that NOLC1 was lowly expressed in ccRCC than that in normal renal tissues, and low expression of NOLC1 predicted the poor survival of patients with ccRCC. Moreover, knockdown of NOLC1 with siRNA could increase the proliferation and migration abilities of ccRCC cells in vitro. Therefore, NOLC1 suppresses cancer progression through various mechanisms in different types of cancers.

Recently, dysregulation of Runx2 expression has been confirmed in various cancers, such as colorectal^28^, thyroid cancer^29^ and breast cancer^40^. These studies indicated that the expression of Runx2 in cancer cells could be regulated by lncRNA, miRNA, and proteins. For instance, lncRNA SNHG3 binding to miR-539 upregulates the expression of Runx2, thereby promotes colorectal cell growth and metastasis^28^. In breast cancer, miR-153 reduces tumor growth and metastasis via direct targeting of Runx2 (ref. ^40^). In this study, we demonstrated that the transcriptional factor Zic2 could promote the expression of Runx2 in ccRCC cells. Consistent with Runx2, Zic2 was highly expressed in ccRCC tissues compared to normal renal tissues, indicating the worse outcome of ccRCC patients. Moreover, increased Zic2 expression was also obviously related to poor survival of patients with nasopharyngeal carcinoma^41^, cervical cancer^42^, and HCC^43^. Knockdown of Zic2 inhibited the EMT process in prostate cancer cells by reducing the level of β-catenin^44^. In addition, β-catenin signaling was capable of boosting the expression of Runx2 in bone differentiation^45^. Therefore, Zic2 may upregulate the expression of Runx2 in ccRCC via β-catenin signaling.

Taken together, our study clearly investigates the expression levels, clinical significances, and functions of Runx2, and identifies its upstream regulator Zic2 and downstream target NOLC1 in ccRCC. Multi-predicators including increased Zic2–Runx2 and downregulated NOLC1 may service as a promising prognostic factor for ccRCC patients. Zic2/Runx2/NOLC1 signaling axis enhances ccRCC cell growth and metastasis, indicating a set of potential therapeutic targets.

## Supplementary information

Supplementary Figure Legends

Figure S1

Figure S2

Figure S3

Figure S4

Figure S5

Figure S6

Table S1

Table S2
