# Supplementary material for: A Zic2/Runx2/NOLC1 signaling axis mediates tumor growth and metastasis in clear cell renal cell carcinoma
Source: Cell Death Dis. 2021 Mar 25;12(4):319. doi: 10.1038/s41419-021-03617-8 (PMC7994417; doi:10.1038/s41419-021-03617-8)
Supplement: Supplementary file 1 — Supplementary Figure Legends [file 41419_2021_3617_MOESM1_ESM.docx]

**Supplementary Figure Legends**

**Figure S1. Log-rank test showed the poor disease free survival of ccRCC patients with high Runx2.**

**Figure S2. Runx2 inhibit the expression of NOLC1 in ccRCC.**

**(A-B)** The binding site of Runx2 in the promoter of NOLC1. **(C)** ChIP-qPCR analysis showed that Runx2 bound to the promoter of NOLC1 in ACHN and 786-O cells. **(D)** TCGA data analysis indicated the low mRNA expression of NOLC1 in ccRCC tissues than that in normal renal tissues (*P* < 0.01). **(E)** IHC staining with antibody against NOLC1 in normal renal and ccRCC tissues. Scale bar = 20 μm in magnified images.

**Figure S3. Promoter methylation (A) and copy number (B) analyses of Runx2 in TCGA database.**

**Figure S4. Zic2 up-regulates Runx2 in ccRCC.**

**(A)** Correlation analysis of Runx2 and Zic2 genes mRNA levels in ccRCC was performed using TCGA cohort (*P* < 0.001). **(B)** IHC staining with antibody against Zic2, Runx2 and NOLC1 in serial ccRCC tissue sections. Scale bar, 50 μm. **(C)** qPCR analysis showed that knockdown of Zic2 reduced the expression of Runx2 in 786-O cell. **(D)** TCGA data analysis suggested the high expression of Zic2 in ccRCC than that in normal renal tissues (*P* < 0.001). **(E)** IHC staining in ccRCC tissue microarray indicated the up-regulation of Zic2 in ccRCC, compared to normal renal tissues (*P* < 0.001).

**Figure S5. qRT–PCR analysis showed that knockdown of Zic2 or Runx2 down-regulated the level of 45S pre-rRNA in 786-O cells.**

**Figure S6.** **Kaplan-Meier survival analyses of ccRCC patients based on the expression level of Zic2/Runx2/NOLC1 signaling.**

**(A)** High expression of both Zic2 and Runx2 predicted shorter overall survival time of ccRCC patients. **(B)** Survival curves suggested the worse outcome of ccRCC patients with high Zic2/Runx2 and low NOLC1.
