# Supplementary figures and images for: A Zic2/Runx2/NOLC1 signaling axis mediates tumor growth and metastasis in clear cell renal cell carcinoma
Source: Cell Death Dis. 2021 Mar 25;12(4):319. doi: 10.1038/s41419-021-03617-8 (PMC7994417; doi:10.1038/s41419-021-03617-8)

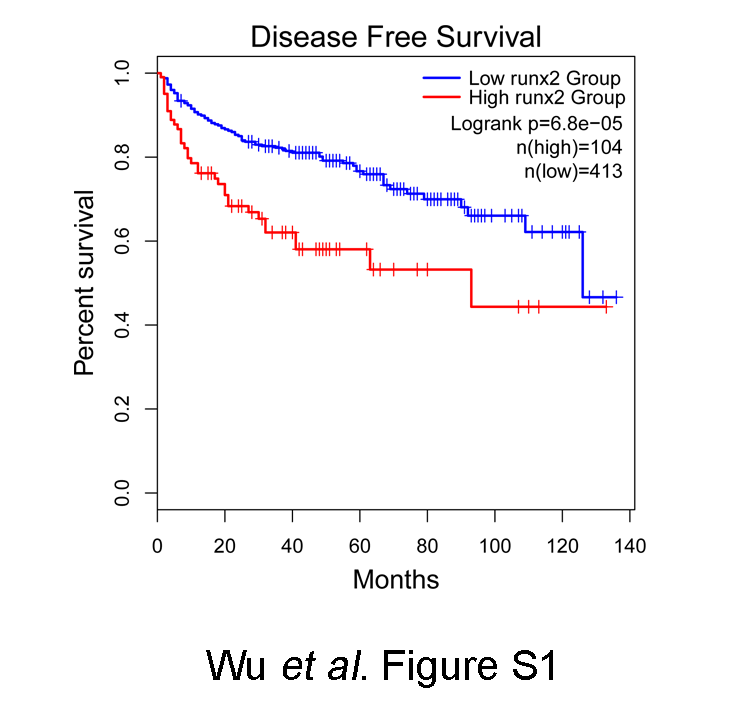

Supplement: Supplementary file 2 — Figure S1 [file 41419_2021_3617_MOESM2_ESM.tif]

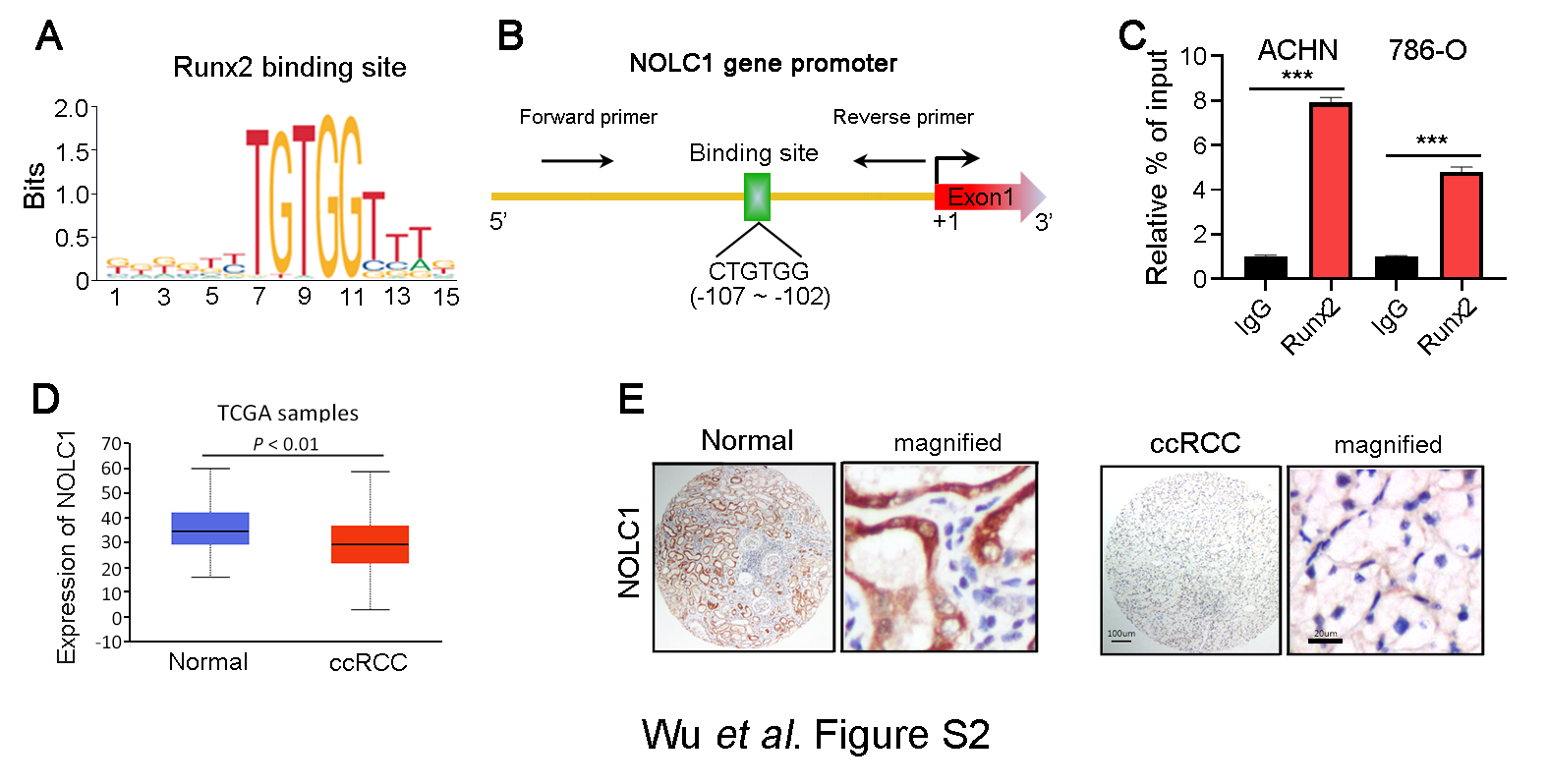

Supplement: Supplementary file 3 — Figure S2 [file 41419_2021_3617_MOESM3_ESM.tif]

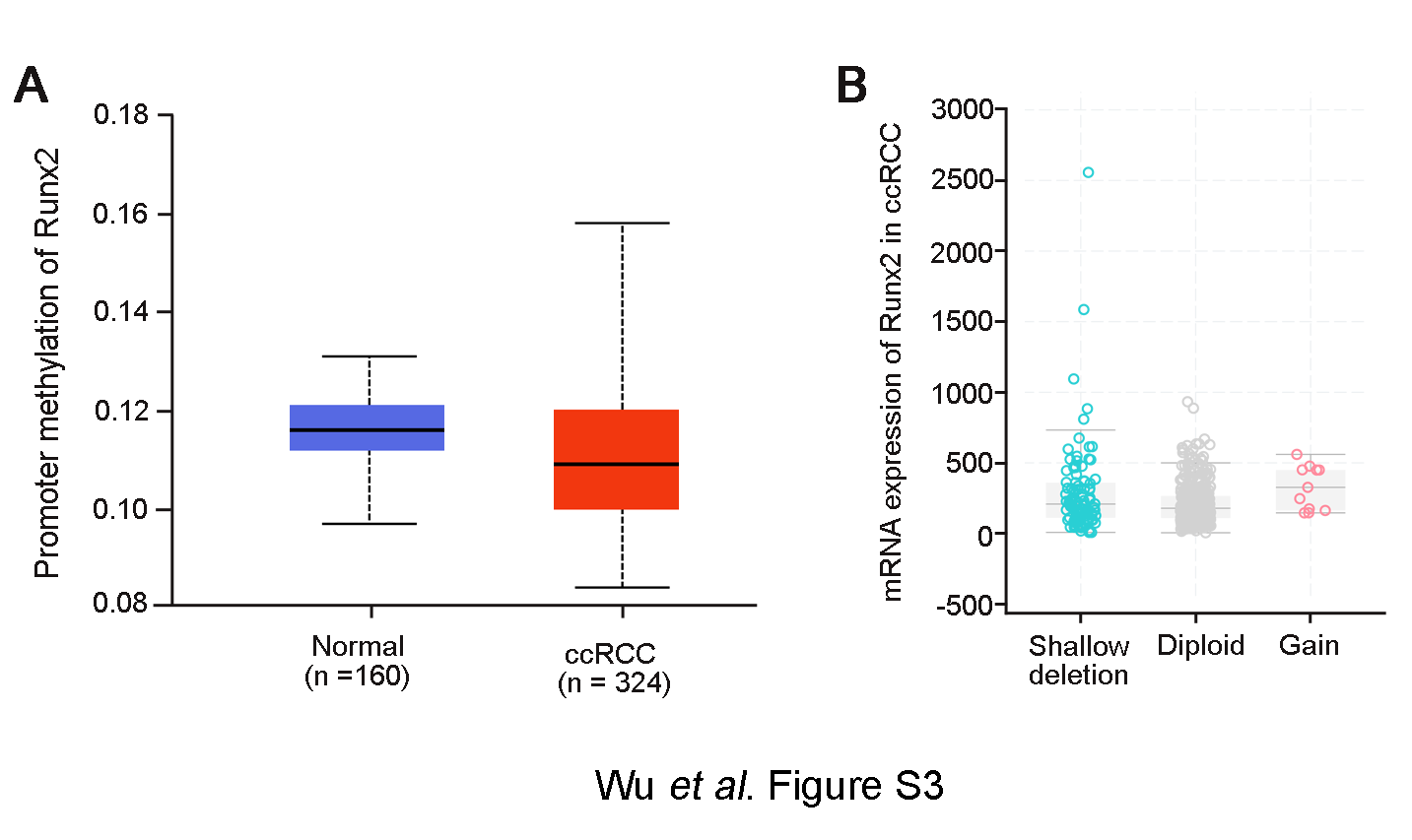

Supplement: Supplementary file 4 — Figure S3 [file 41419_2021_3617_MOESM4_ESM.tif]

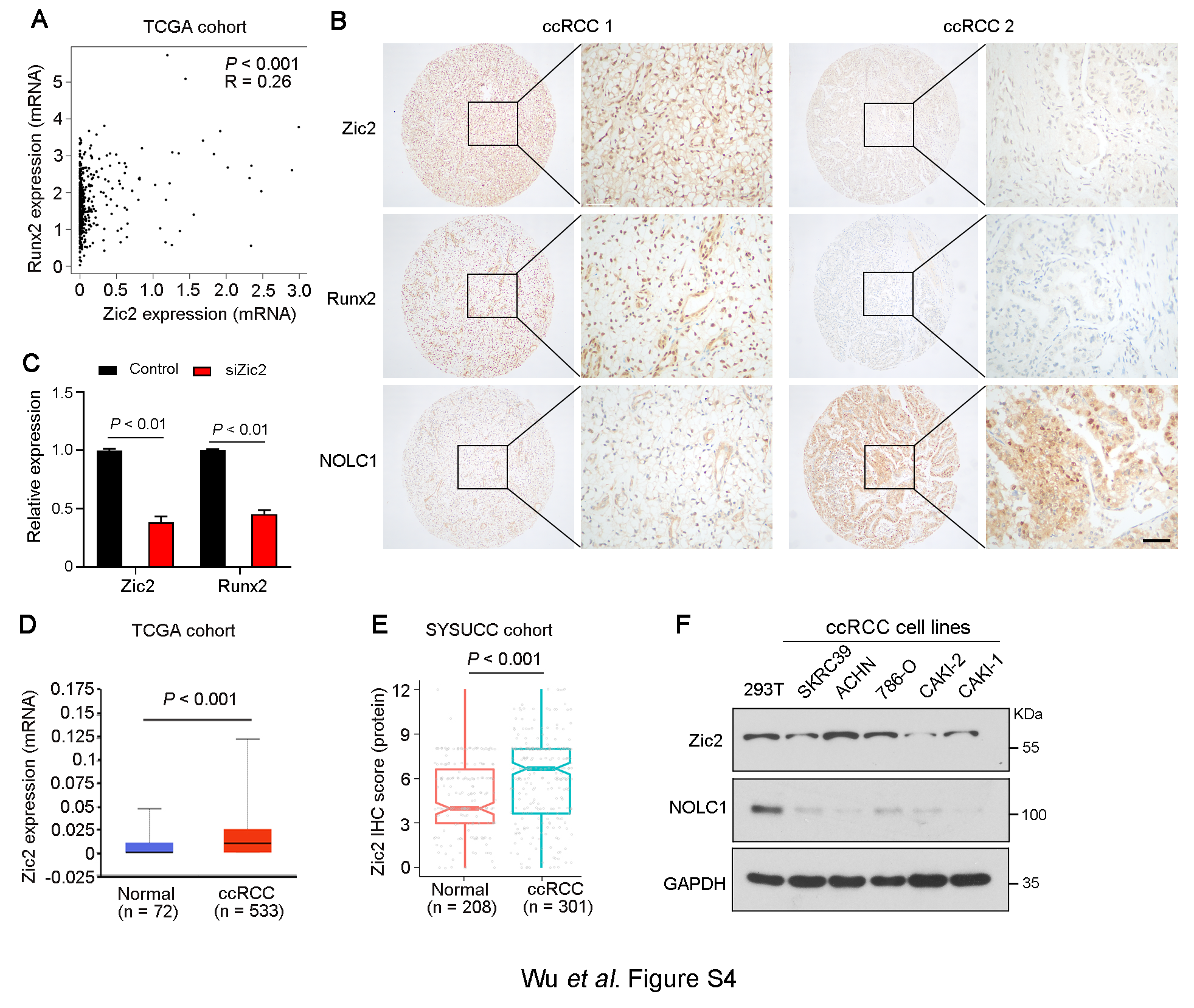

Supplement: Supplementary file 5 — Figure S4 [file 41419_2021_3617_MOESM5_ESM.tif]

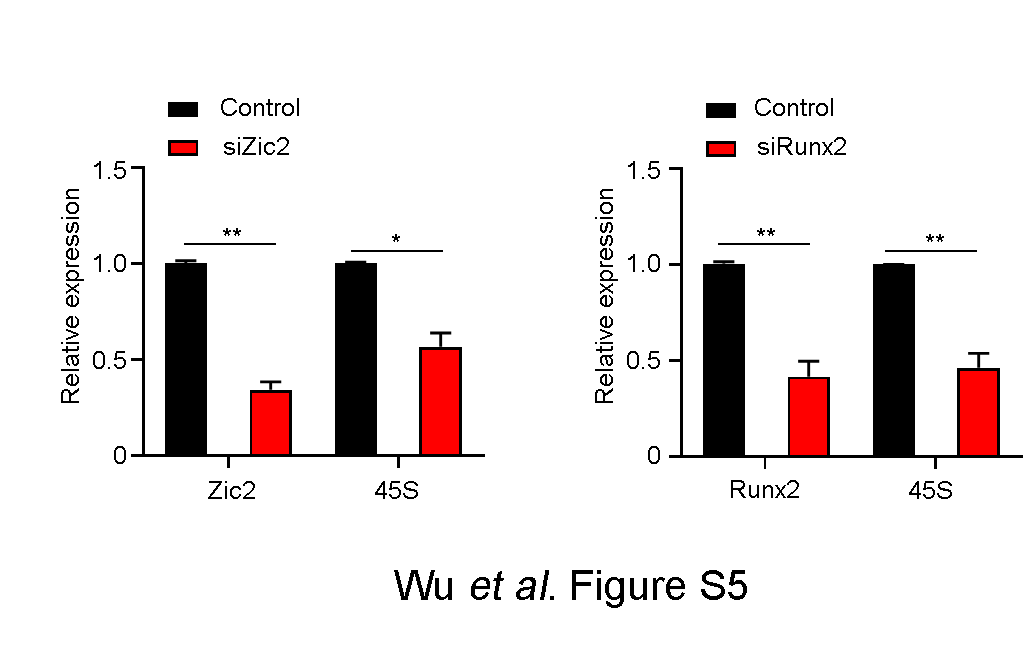

Supplement: Supplementary file 6 — Figure S5 [file 41419_2021_3617_MOESM6_ESM.tif]

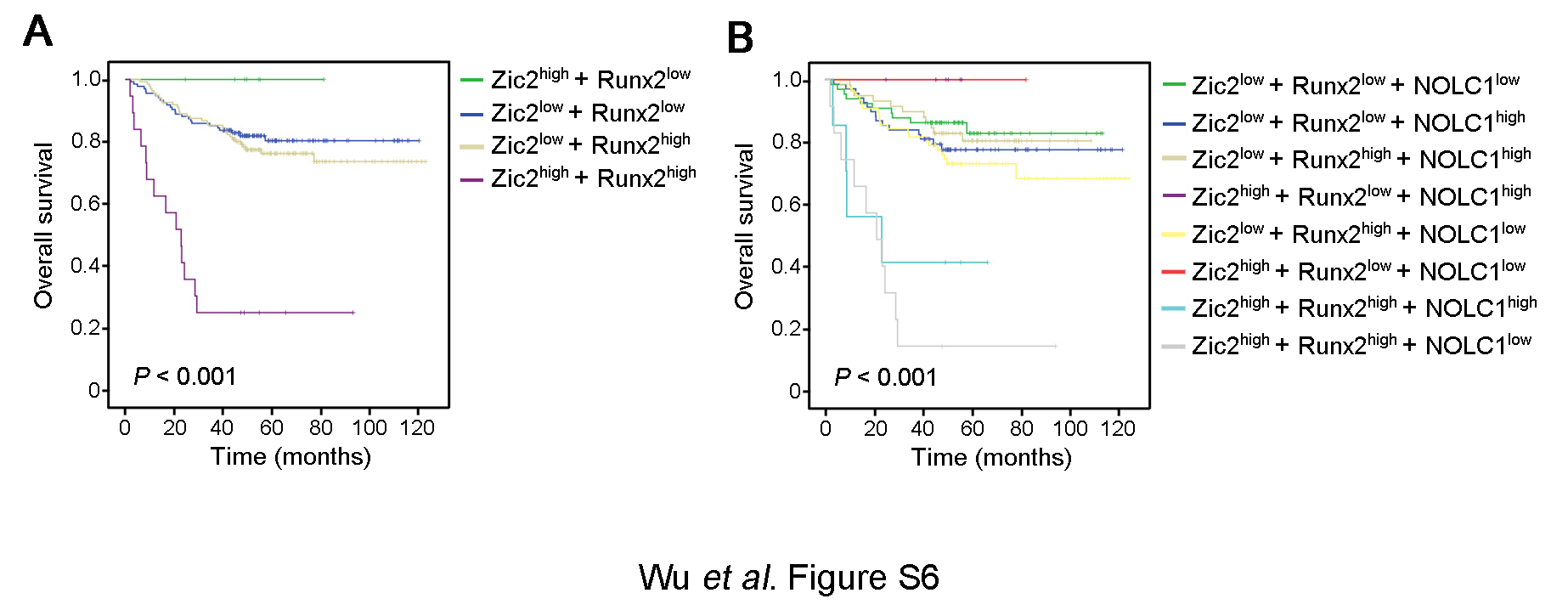

Supplement: Supplementary file 7 — Figure S6 [file 41419_2021_3617_MOESM7_ESM.tif]
