# Supplementary material for: A Zic2/Runx2/NOLC1 signaling axis mediates tumor growth and metastasis in clear cell renal cell carcinoma
Source: Cell Death Dis. 2021 Mar 25;12(4):319. doi: 10.1038/s41419-021-03617-8 (PMC7994417; doi:10.1038/s41419-021-03617-8)
Supplement: Supplementary file 8 — Table S1 [file 41419_2021_3617_MOESM8_ESM.docx]

**Table S1 siRNA used for RNA silencing**

| **siRNA** | **Sequence (5'-3')** |
| --- | --- |
| Control | UUCUCCGAACGUGUCACG |
| Zic2#1 | CUGUGUACAUAGCGGACUC |
| Zic2#2 | AACUCCGGAUUGCGUUCCU |
| Runx2#1 | CCAGCCACCUUUACUUACA |
| Runx2#2 | GAAGCUUGAUGACUCUAAA (also used for shRNA) |
| NOLC1#1 | CACCAAGAAUUCUUCAAAU |
| NOLC1#2 | GAGCUCUGAUUCUGAUUCUT |
