# Supplementary material for: A Zic2/Runx2/NOLC1 signaling axis mediates tumor growth and metastasis in clear cell renal cell carcinoma
Source: Cell Death Dis. 2021 Mar 25;12(4):319. doi: 10.1038/s41419-021-03617-8 (PMC7994417; doi:10.1038/s41419-021-03617-8)
Supplement: Supplementary file 9 — Table S2 [file 41419_2021_3617_MOESM9_ESM.docx]

**Table S2 Primers used for qRT-PCR.**

| **Genes** | **Primer sequence (5'-3')** |
| --- | --- |
| Zic2 | Forward: CACCTCCGATAAGCCCTATCT |
|  | Reverse: GGCGTGGACGACTCATAGC |
| Runx2 | Forward: CCGCCTCAGTGATTTAGGGC |
|  | Reverse: GGGTCTGTAATCTGACTCTGTCC |
| NOLC1 | Forward: TTCCTGCGCGATAACCAACTC |
|  | Reverse: CCTGTAACTTTCGCTCTGGGA |
| 18S | Forward: TGAGAAACGGCTACCACATCC |
|  | Reverse: ACCAGACTTGCCCTCCAATG |
| 45S | Forward: GTGTGTGGGTTGACTTCGGA |
|  | Reverse: AAGGCTTTTCTCACCGAGGG |
| NOLC1 promoter | Forward: TCTGAGGTGAAGGTGGGACT |
|  | Reverse: GGCCTGTGTTACGTCATTGC |
